# Realization of a cold atom gyroscope in space

**DOI:** 10.1093/nsr/nwaf012

**Published:** 2025-01-11

**Authors:** Jinting Li, Xi Chen, Danfang Zhang, Wenzhang Wang, Yang Zhou, Meng He, Jie Fang, Lin Zhou, Chuan He, Junjie Jiang, Huanyao Sun, Qunfeng Chen, Lei Qin, Xiao Li, Yibo Wang, Xiaowei Zhang, Jiaqi Zhong, Runbing Li, Meizhen An, Long Zhang, Shuquan Wang, Zongfeng Li, Jin Wang, Mingsheng Zhan

**Affiliations:** State Key Laboratory of Magnetic Resonance and Atomic and Molecular Physics, Division of Precision Measurement Physics, Innovation Academy for Precision Measurement Science and Technology, Chinese Academy of Sciences, Wuhan 430071, China; School of Physical Sciences, University of Chinese Academy of Sciences, Beijing 100049, China; State Key Laboratory of Magnetic Resonance and Atomic and Molecular Physics, Division of Precision Measurement Physics, Innovation Academy for Precision Measurement Science and Technology, Chinese Academy of Sciences, Wuhan 430071, China; State Key Laboratory of Magnetic Resonance and Atomic and Molecular Physics, Division of Precision Measurement Physics, Innovation Academy for Precision Measurement Science and Technology, Chinese Academy of Sciences, Wuhan 430071, China; School of Physical Sciences, University of Chinese Academy of Sciences, Beijing 100049, China; State Key Laboratory of Magnetic Resonance and Atomic and Molecular Physics, Division of Precision Measurement Physics, Innovation Academy for Precision Measurement Science and Technology, Chinese Academy of Sciences, Wuhan 430071, China; School of Physical Sciences, University of Chinese Academy of Sciences, Beijing 100049, China; State Key Laboratory of Magnetic Resonance and Atomic and Molecular Physics, Division of Precision Measurement Physics, Innovation Academy for Precision Measurement Science and Technology, Chinese Academy of Sciences, Wuhan 430071, China; School of Physical Sciences, University of Chinese Academy of Sciences, Beijing 100049, China; State Key Laboratory of Magnetic Resonance and Atomic and Molecular Physics, Division of Precision Measurement Physics, Innovation Academy for Precision Measurement Science and Technology, Chinese Academy of Sciences, Wuhan 430071, China; State Key Laboratory of Magnetic Resonance and Atomic and Molecular Physics, Division of Precision Measurement Physics, Innovation Academy for Precision Measurement Science and Technology, Chinese Academy of Sciences, Wuhan 430071, China; State Key Laboratory of Magnetic Resonance and Atomic and Molecular Physics, Division of Precision Measurement Physics, Innovation Academy for Precision Measurement Science and Technology, Chinese Academy of Sciences, Wuhan 430071, China; Hefei National Laboratory, Hefei 230088, China; State Key Laboratory of Magnetic Resonance and Atomic and Molecular Physics, Division of Precision Measurement Physics, Innovation Academy for Precision Measurement Science and Technology, Chinese Academy of Sciences, Wuhan 430071, China; State Key Laboratory of Magnetic Resonance and Atomic and Molecular Physics, Division of Precision Measurement Physics, Innovation Academy for Precision Measurement Science and Technology, Chinese Academy of Sciences, Wuhan 430071, China; School of Physical Sciences, University of Chinese Academy of Sciences, Beijing 100049, China; State Key Laboratory of Magnetic Resonance and Atomic and Molecular Physics, Division of Precision Measurement Physics, Innovation Academy for Precision Measurement Science and Technology, Chinese Academy of Sciences, Wuhan 430071, China; State Key Laboratory of Magnetic Resonance and Atomic and Molecular Physics, Division of Precision Measurement Physics, Innovation Academy for Precision Measurement Science and Technology, Chinese Academy of Sciences, Wuhan 430071, China; State Key Laboratory of Magnetic Resonance and Atomic and Molecular Physics, Division of Precision Measurement Physics, Innovation Academy for Precision Measurement Science and Technology, Chinese Academy of Sciences, Wuhan 430071, China; State Key Laboratory of Magnetic Resonance and Atomic and Molecular Physics, Division of Precision Measurement Physics, Innovation Academy for Precision Measurement Science and Technology, Chinese Academy of Sciences, Wuhan 430071, China; State Key Laboratory of Magnetic Resonance and Atomic and Molecular Physics, Division of Precision Measurement Physics, Innovation Academy for Precision Measurement Science and Technology, Chinese Academy of Sciences, Wuhan 430071, China; State Key Laboratory of Magnetic Resonance and Atomic and Molecular Physics, Division of Precision Measurement Physics, Innovation Academy for Precision Measurement Science and Technology, Chinese Academy of Sciences, Wuhan 430071, China; State Key Laboratory of Magnetic Resonance and Atomic and Molecular Physics, Division of Precision Measurement Physics, Innovation Academy for Precision Measurement Science and Technology, Chinese Academy of Sciences, Wuhan 430071, China; Hefei National Laboratory, Hefei 230088, China; State Key Laboratory of Magnetic Resonance and Atomic and Molecular Physics, Division of Precision Measurement Physics, Innovation Academy for Precision Measurement Science and Technology, Chinese Academy of Sciences, Wuhan 430071, China; Hefei National Laboratory, Hefei 230088, China; Department of Quantum Perception Research, Wuhan Institute of Quantum Technology, Wuhan 430206, China; Laboratory of Space Experimental Technology, Technology and Engineering Center for Space Utilization, Chinese Academy of Sciences, Beijing 100094, China; Laboratory of Space Experimental Technology, Technology and Engineering Center for Space Utilization, Chinese Academy of Sciences, Beijing 100094, China; Laboratory of Space Experimental Technology, Technology and Engineering Center for Space Utilization, Chinese Academy of Sciences, Beijing 100094, China; Laboratory of Space Experimental Technology, Technology and Engineering Center for Space Utilization, Chinese Academy of Sciences, Beijing 100094, China; State Key Laboratory of Magnetic Resonance and Atomic and Molecular Physics, Division of Precision Measurement Physics, Innovation Academy for Precision Measurement Science and Technology, Chinese Academy of Sciences, Wuhan 430071, China; Hefei National Laboratory, Hefei 230088, China; Department of Quantum Perception Research, Wuhan Institute of Quantum Technology, Wuhan 430206, China; State Key Laboratory of Magnetic Resonance and Atomic and Molecular Physics, Division of Precision Measurement Physics, Innovation Academy for Precision Measurement Science and Technology, Chinese Academy of Sciences, Wuhan 430071, China; Hefei National Laboratory, Hefei 230088, China; Department of Quantum Perception Research, Wuhan Institute of Quantum Technology, Wuhan 430206, China

**Keywords:** atom interferometer, space, microgravity, gyroscope

## Abstract

High-precision gyroscopes in space are essential for fundamental physics research and navigation. Due to its potential high precision, the cold atom gyroscope is expected to be one of the next generation of gyroscopes in space. Here, we report the first realization of a cold atom gyroscope, which was demonstrated by the atom interferometer installed in the China Space Station (CSS) as a payload. By compensating for the CSS's high dynamic rotation rate using a built-in piezoelectric mirror, spatial interference fringes in the interferometer are successfully obtained. Then, the optimized ratio of the Raman laser's angles is derived, the coefficients of the piezoelectric mirror are self-calibrated in orbit, and various systemic effects are corrected. We achieve a rotation measurement resolution of 50 μrad/s for a single shot and 17 μrad/s for an average number of 32. The measured rotation is −1142 ± 29 μrad/s and is compatible with that recorded by the classical gyroscope of the CSS. This study paves the way for developing high-precision cold atom gyroscopes in space.

## Teaser

This work demonstrates the first realization of an in-orbit cold atom gyroscope using the China Space Station Atom Interferometer, representing the emergence of a space quantum inertial sensing epoch.

## INTRODUCTION

Space-based gyroscopes are important in inertial navigation and fundamental physics tests [1–5]. One typical example is the Gravity Probe B (GP-B) satellite, which utilizes cryogenic gyroscopes to test general relativity. The GP-B project gives a test precision of the frame-dragging effect of 19% with one year of measurement data [3]. No violation was observed for this general relativity effect from its theoretical prediction. Further experiments with the Laser Relativity Satellite 2 (LARES 2) [6] in space and Gyroscopes IN General Relativity (GINGER) [7] on the ground will help to continuously improve the test precision.

Atom interferometers (AIs) are expected to be next-generation gyroscopes for measuring rotation with very high precision, as already demonstrated on the ground [8–11]. In space, AIs could achieve a much longer interference time than those on the ground, thus forming a space gyroscope with precision comparable to the cryogenic gyroscope. For example, the Hyper project is designed to have a rotation measurement resolution of 10^−12^ rad/s/$\sqrt {{\mathrm{Hz}}} $, and aims to test the frame-dragging effect with a precision of 10% [12]. Besides, cold atom gyroscopes with such high precision will improve the precision of inertial navigation, especially when the Global Navigation Satellite System (GNSS) signal is unavailable or for deep space exploration [13]. Early studies have been carried out for interference experiments under microgravity platforms such as the drop tower [14,15], sounding rocket [16,17], parabolic flying plane [18,19] and the International Space Station [20–22]. However, in-orbit rotation measurement by an AI has not been realized up to now.

There are several challenges to realizing the cold atom gyroscope in space. First, in microgravity and under a retroreflector Raman transition configuration, the energy levels of the Raman transitions of the two Raman laser pairs are degenerate. This will automatically form the double diffraction interference loop. How to use this interference configuration to measure the rotation has not been explored yet and needs to be verified. Second, in space, the rotation rate is usually much higher than the Earth's rotation rate. How to precisely extract the rotation in such a high dynamic condition without losing contrast still needs investigation. Third, the distribution of the cold atom cloud will induce loss of contrast and variation of the spatial frequency for the interference fringe. Finding a proper scheme to eliminate this effect is important for improving the rotation measurement precision.

In this article, we report the first rotation measurement result using a compact AI payload in the China Space Station (CSS). The point source interferometry (PSI) method [23,24] based on double diffraction Raman transition is realized under a microgravity environment. Spatial interference fringes are obtained and modulated using a built-in piezoelectric mirror, and rotation and acceleration are extracted from the interference fringes. This device is critical for precisely compensating the rotation rate of CSS, which is 15-fold higher than the Earth's rotation rate. The complete expression of the rotation-induced phase, including the effects of the Raman laser's angles and the distributions of the cold atom cloud, is derived to measure the rotation. The ratio of the Raman laser's angles is optimized to eliminate the decoherent effect caused by the cold atom's position and velocity distribution, and these angles are self-calibrated in orbit to be better than 1 μrad by using the PSI fringes. Finally, we achieve a rotation measurement resolution of 50 μrad/s for a single shot and a long-term stability of 17 μrad/s for an average number of 32. After the systemic error correction, the measured rotation value is −1142 ± 29 μrad/s, which agrees well with that measured by the classical gyroscope of the CSS platform. This work achieves the first AI-based gyroscope in space. The measurement scheme, the error estimation method and the engineering design lay a foundation for the further development of cold atom gyroscopes in space.

## RESULTS

### Atom interference process of CSSAI

**Figure 1.**
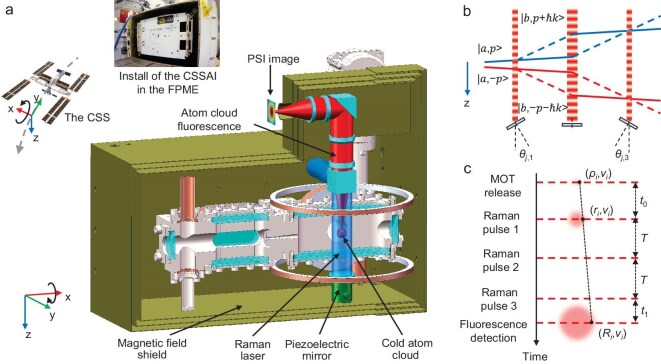
The working principle of the China Space Station Atom Interferometer (CSSAI). (a) The China Space Station (CSS), the installed CSSAI in the Free-floating Platform for Microgravity Experiment (FPME), and the CSSAI's physical system profile. The cold atom cloud, the Raman laser for the point source interferometry (PSI) and the imaging of the fluorescence of the cold atom cloud are also illustrated in the physical system. (b) The double single diffraction (DSD) Raman transition and Raman interference loop scheme for the ^87^Rb atom. The states |a > and |b > represent the |5^2^*S*_1/2_, *F* = 1, *M_F_* = 0 > and |5^2^*S*_1/2_, *F* = 2, *M_F_* = 0 > states. (c) Atom position changes over time during the interference experiment.

The China Space Station Atom Interferometer (CSSAI) is an integrated ^85^Rb-^87^Rb dual-species AI payload. It has a size of 46 cm × 33 cm × 26 cm and a maximum power consumption of ∼75 W. After the ground functional test, this payload was launched to the CSS in November 2022. The installation of the CSSAI in the CSS is shown in Fig. 1a. Supplementary Data section I describes a brief outline of the payload and its operation. More details can be found in ref. [25].

The atom interference process of CSSAI is shown in Fig. 1b. ^87^Rb atom clouds with more than 10^8^ atoms with a temperature of 2 μK are produced using a 2D magneto-optical trap (2D-MOT) and 3D magneto-optical trap (3D-MOT). The atoms are then optically pumped from the |5^2^*S*_1/2_, *F* = 2 > state to the |5^2^*S*_1/2_, *F* = 1 > state. Because of the microgravity, the released cold atom cloud is at the center of the 3D-MOT chamber, and its center velocity is zero. A linear polarization Raman laser is transmitted through a quarter wave plate and reflected by a mirror to produce the perpendicular linear polarization Raman laser pair. For the zero-velocity atom, the Raman laser pair will drive two Raman transitions in opposite directions, and the two-photon detuning of the Raman transitions will be the same. This will automatically induce the double diffraction Raman transition [19,26]. Here, we apply the double single diffraction (DSD) Raman transition [19] to create two symmetrical M-Z interference loops composed of |5^2^*S*_1/2_, *F* = 2, *M_F_* = 0 > state and |5^2^*S*_1/2_, *F* = 1, *M_F_* = 0 > state, as shown in Fig. 1b. The duration of the Raman π pulse is set to 17 μs, and the two-photon detuning is set to 74 kHz to select two groups of cold atoms with opposite velocity. Because of the microgravity, the Doppler shift of the Raman transitions is nearly constant, and the Raman laser's frequency chirp is not introduced. A closed-loop two-axis piezoelectric mirror is used to control the angle of the Raman laser during interference. This device is critical to inducing the PSI and creating the spatial interference fringe. After the interference, atoms in the |5^2^*S*_1/2_, *F* = 2 > state are fluorescently excited. The Materials and Methods section provides a more detailed description of the time sequence and various parameters during the interference. The fluorescence passes through a polarization beam splitter and is imaged by a scientific Complementary Metal Oxide Semiconductor (CMOS) camera in the same direction as the Raman laser, as shown in Fig. 1a. The imaging system has a magnification factor of 2.22 ± 0.03, which is described in Supplementary Data section II. To enhance the signal-to-noise ratio of the spatial interference fringe, the fluorescence image is averaged in a 1D curve, and a normalization method is designed to eliminate this background and normalize the spatial fringe's bias and amplitude fluctuation, which is introduced in Supplementary Data section III. Sine fitting is used to extract the fringe's phase and spatial frequency. The principal component analysis (PCA) method is also used to calculate the principal components for different orders.

### Derivation and optimization of the phase expression of PSI

Considering the acceleration, rotation and Raman laser's angle, the spatial phase of the PSI [27] can be expressed as


(1) \begin{eqnarray*}
\phi &=& {k_{{\mathrm{eff}}}}{a_z}{T^2}\\
&&\!\!\! + \mathop \sum \limits_i {\delta _i}{k_{{\mathrm{eff}}}}[ {2{\Omega _j}{v_i}{T^2}\! +\! {\theta _{j,1}}{r_i}\! +\! {\theta _{j,3}}\left( {{r_i}\! +\! 2{v_i}T} \right)} ], \\
\end{eqnarray*}

where *k*_eff_ is the effective wave vector, *T* is the time interval between the Raman pulses, *i* represents the *x* and *y* coordinate, and *j* is the opposite of *i*. The symbol *δ_i_* is defined as *δ_x_* = 1 and *δ_y_* = −1, *a_z_* is the residual acceleration in the *z*-axes, *Ω_j_* is the rotation of CSS in the *j*-axes and *θ_j_*_,1_ and *θ_j_*_,3_ are the angles of the Raman laser in the *j*-axes at the time of the first and third Raman laser pulses relative to the angle at the time of the second Raman laser pulse. *r_i_* and *v_i_* are the position and velocity of the atom at the time of the first Raman laser pulse. The spatial phase also includes terms of the square of ***Ω***, the time derivation of ***Ω***, the gravity gradient, and their coupling. The terms of the square of ***Ω*** and the gravity gradient contribute to *a_z_* and do not affect the rotation measurement. The term of the time derivation of ***Ω*** and the coupling term are 6–8 orders of magnitude smaller than other terms and are safely omitted. Supplementary Data section IV gives the detailed derivation. The imaging process will project the 3D population of the atom cloud to the 2D imaging plane. Because the phase variation and imaging plane are both in the x-y plane, the formula of the phase of the 2D spatial fringe is the same as that of Equation (1).

The phase is related to *r_i_* and *v_i_*. However, for the fluorescence image, what we measured was the spatial population of the atom at the fluorescence detection time, as shown in Fig. 1c. One has *R_i_* = *r_i_* + *v_i_*(2*T* + *t*_1_), where *R_i_* is the atom's position at the fluorescence detection time and *t*_1_ is the time interval between the third Raman laser pulse and the fluorescence excitation laser pulse. For general cases, the position and velocity distribution of the atom will induce decoherence and period variation for the spatial fringe [28,29]. As simulated in Supplementary Data section V and Fig. S7. If we submit *r_i_* = *R_i_* − *v_i_*(2*T* + *t*_1_) in Equation (1) and let the coefficient of *v_i_* be zero we will have the following relationship:


(2) \begin{eqnarray*}
{\theta _{j,1}} = {\theta _{jo,1}} = \frac{{ - {t_1}{\theta _{j,3}} + 2\,\,{\Omega _j}\,\,{T^2}}}{{2\,\,T + {t_1}}}.
\end{eqnarray*}

We define *θ_jo_*_,1_ as the optimized angle. Then, *ϕ* is only related to *R_i_*. Both the effects of the offset and distributions of the position and velocity of the atom are eliminated, and the contrast of the fringe will be maximized. The optimized phase has the following form:


(3) \begin{eqnarray*}
{\phi _o} = {k_{{\mathrm{eff}}}}{a_z}{T^2} + \mathop \sum \limits_{i = x,y} {\delta _i}{f_{io}}{R_i}
\end{eqnarray*}

(4) \begin{eqnarray*}
{f_{io}} = \frac{{2{k_{{\mathrm{eff}}}}}}{{2T + {t_1}}}\left( {{\theta _{j,3}}T + {\Omega _j}{T^2}} \right),
\end{eqnarray*}

where *f_io_* is defined as the optimized spatial frequency of the spatial fringe.

For a more general case where Equation (2) is not fulfilled, the exact expression for the phase and the spatial frequency is derived. By considering the atom cloud's position and velocity distributions and integrating the phase of Equation (1) over them, strict analytical formulas for the phase and the spatial frequency are derived in the Materials and Methods section as illustrated by Equations (8) and (9). The exact solution of the spatial frequency is additionally related to the differential angle Δ*θ_j_* = *θ_j_*_,1_ − *θ_jo_*_,1_ and the width of the distributions of the atom cloud. So, by setting or measuring the various parameters in Equation (9), and fitting the spatial frequency from the interference fringe, the rotation value can be calculated.

### In-orbit calibration of the Raman laser's angles

The spatial frequency is related to the angle of the Raman laser, which is controlled by the piezoelectric mirror. The angle is proportional to the control voltage of the piezoelectric mirror with the relationship *θ_j_* = *α_j_V_j_*, where *α_j_* is defined as the voltage-angle coefficient. This coefficient is calibrated on the ground. However, the calibrated piezoelectric mirror is installed into the physical system and passes the mechanical and thermal tests. The coefficient might have some change. The accuracy angle is critical for calculating the rotation. However, one cannot calibrate it with external equipment after it is installed in the payload, especially after the payload is installed in the CSS.

**Figure 2.**
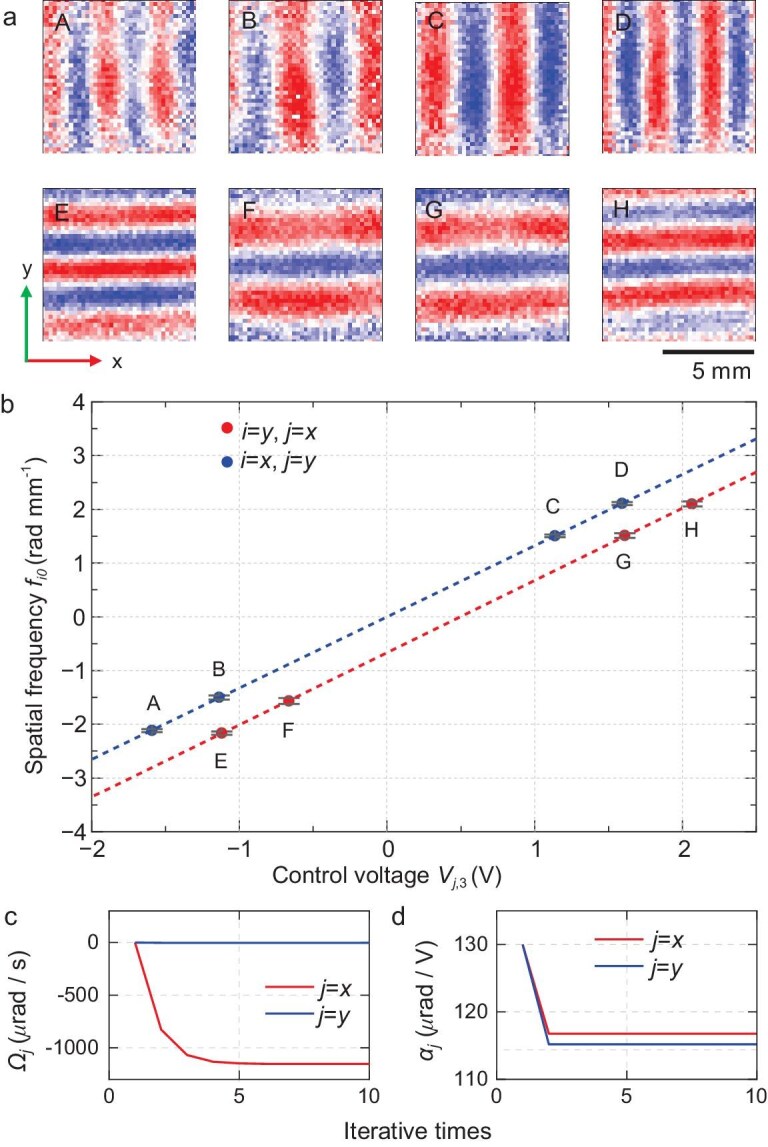
Calibration of the Raman laser's angle. (a) First-order principal images of the 2D spatial interference fringes for the measurement points in Fig. 2b. (b) The relationship between the measured spatial frequencies *f_i_* and the control voltages of the piezoelectric mirror *V_j_*_,3_. The dashed lines are the corresponding linear fitting curves. (c and d) The variation of the measured rotation values *Ω_j_* and the voltage-angle coefficients *α_j_* during the iterative process.

Here, a self-calibration method is proposed and realized to measure the coefficient precisely by carrying out the in-orbit PSI experiment with *T* = 50 ms and *t*_1_ = 40 ms. From Equation (4), one can see that the optimized spatial frequency is proportional to the angle *θ_j_*_,3_, and thus proportional to *V_j_*,_3_. By setting a group value of *V_j_*,_3_, adjusting the value of *V_j_*,_1_ to satisfy the relationship of Equation (2), and carrying out the PSI experiment to measure the spatial frequency *f_io_*, as shown in Fig. 2, the coefficient *α_j_* could be obtained by linearly fitting the values of *f_io_* and *V_j_*,_3_. However, for an inaccurate value of *α_j_*, the calculated angles *θ_j_* are inaccurate too, and Equation (2) holds approximatively. The exact spatial frequency *f_i_* should be calculated by Equation (9), where *f_i_* is not proportional to *V_j_*,_3_ strictly.

To solve this problem, an iterative method is applied to get the exact value of *α_j_*. First, we set an initial set of estimated values of *α_j_* and *Ω_j_*, calculate the differential angle Δ*θ_j_* by using Equation (2), and calculate the differential spatial frequency Δ*f_j_* by using Equation (9). Then *f_i_* is subtracted by Δ*f_j_* to obtain *f_io_*. The values of *f_io_* and *V_j_*,_3_ are linearly fitted to get a new set of values of *α_j_* and *Ω_j_*. This production continues until both *α_j_* and *Ω_j_* converge. The convergence process of *Ω_j_* and *α_j_* and the final convergent fitting curves for *f_io_* are shown in Fig. 2c, d and b. The calculated coefficients are *α_x_* = 116.75 ± 0.41 μrad/V and *α_y_* = 115.21 ± 0.20 μrad/V respectively. From the obtained voltage-angle coefficients, the angle control precision is estimated to be 0.85 μrad and 0.33 μrad in the x and y directions. The obtained values of rotation are *Ω_x_* = −1153 ± 12 μrad/s and *Ω_y_*=−3.7 ± 5.7μrad/s. The significant rotation rate in the x direction is due to the nadir-pointing rotation of the CSS around the Earth.

### Rotation extraction and error estimation

**Figure 3.**
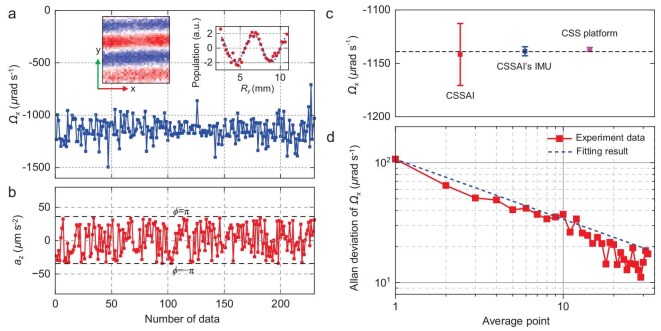
Rotation and acceleration measurement in space. (a) The measured value of the rotation *Ω_x_*. The inset images are the first-order principal images of the 2D spatial interference fringes for the measurement and a typical normalized 1D interference fringe. (b) The measured value of the acceleration *a_z_*. The dashed lines represent the corresponding fitting phase of π and –π. (c) Comparison of the measured rotation value by the CSSAI, CSSAI's inertial measuring unit (IMU) and the classical gyroscope of the CSS platform. The dashed line indicates their average value. (d) The Allan derivation of the measured rotation *Ω_x_*. The dashed line is its fitting curve with white noise.

To measure the rotation *Ω_x_* and acceleration *a_z_* more precisely, PSI experiments with *T* = 75 ms and *t*_1_ = 40 ms are carried out. The phase and spatial frequency are fitted from the fringes, and Equations (8) and (9) are used to calculate the acceleration and rotation. To improve the measurement precision of rotation, the finite pulse width effect of the Raman laser is calculated and corrected (see Materials and Methods for detailed derivation). The calculated results of *Ω_x_* and *a_z_* are shown in Fig. 3a and b. Due to the vibration of the CSS, the acceleration-induced phase variation exceeds 2π, and the definite value for the acceleration is unknown. However, from the fitting residual phase, the measurement resolution of acceleration is estimated to be 1.0 μm/s^2^ for a single shot. The rotation measurement resolution is 50 μrad/s for a single shot. The mean value is 1142 μrad/s, and the standard deviation is 101 μrad/s. The Allan deviation is shown in Fig. 3d, and the measurement resolution is 17 μrad/s for an averaged number of 32.

Rotation measurement errors caused by the parameters’ uncertainties are calculated according to Equation (10) to estimate the measurement precision. Errors caused by the uncertainties of the magnification factor of the imaging system, the Raman laser's angle, the time sequence, the Raman laser frequency and the distribution of the atom cloud are calculated in Supplementary Data section VIA–D. The magnetic-field-gradient-induced spatial frequency error is estimated by the ground measurement result in Supplementary Data section VIE. Other effects that only influence the phase but not the spatial frequency are not considered. These include the residual acceleration, the AC Stack shift and the multi-sideband effect [30,31]. The error terms are listed in Table 1. The largest error terms are the fitting noise of *f_i_*, the uncertainty of the magnification factor and the uncertainty of the Raman laser's angle. The measured rotation value is *Ω_x_* = −1142 ± 29 μrad/s.

**Table 1.** Error analysis for the rotation *Ω_x_* with the PSI method in space.

Parameter terms	Parameter values	Evaluated result (μrad/s)
Spatial frequency (fitting result)	*f_y_* = 1.497 ± 0.013 rad/mm	−1142 ± 17
Magnification factor of the imaging system	*κ*=2.22 ± 0.03	±21
Angles of third Raman laser pulses	*θ_x_*_,3_ = 202.94 ± 0.72 μrad	±10
Difference angle of *θ_x_*_,1_	Δ*θ_x_* = 2.41 ± 0.41 μrad	±1
Interference time	*T* = 75137.3 ± 0.23 μs	±3 × 10^−3^
Time before the first Raman pulse	*t*_0_ = 43245.8 ± 0.13 μs	±2 × 10^−5^
Time after the third Raman laser pulse	*t*_1_ = 40 146 ± 10 μs	±9 × 10^−2^
Width of the Raman π pulse	*τ*=17±(5 × 10^−5^) μs	±6 × 10^−7^
Effective wave vector	*k*_eff_ = 16105813.75 ± 0.09 m^−1^	±9 × 10^−6^
Width of the MOT's position	*σ_ρi_* = 0.427 ± 0.013 mm	±3 × 10^−2^
Width of the MOT's velocity	*σ_vi_* = 14.13 ± 0.18 mm/s	±1 × 10^−2^
Magnetic field	*B*_0_ = 504.7 ± 1.3 mG *γ_i_*_,2_=±1.3 G/m^2^	±2 × 10^−1^
In total		−1142 ± 29

To check the measurement accuracy, we record the rotation measured by the CSSAI's inertial measuring unit (IMU) and the classical gyroscope of the CSS platform for the same time interval. The Micro-Electro-Mechanical System (MEMS) gyroscope of the CSSAI's IMU has an accuracy of 4.8 μrad/s, and the gyroscope of the CSS platform has an accuracy of 1.5 μrad/s. Associating the rotation measurement result of these two gyroscopes and their measurement accuracies, these two gyroscopes give the rotation measurement values of −1138.7 ± 6.3 μrad/s and −1137.0 ± 2.3 μrad/s, respectively. These three measurement values are in good agreement, as shown in Fig. 3c.

## DISCUSSION

This article introduces the integrated AI-based payload in the CSS and reports the first AI-based rotation measurement result in space. Spatial interference fringes are obtained using the PSI method based on the DSD interference scheme. The optimized ratio of the Raman laser's angle is derived to eliminate the decoherent effect caused by the cold atom cloud's position and velocity distribution. Formulas to extract the rotation are derived with the corrections of the offset of the Raman laser's angle and finite Raman laser pulse effect. In-orbit self-calibration of the Raman laser's angle is implemented to improve the accuracy of rotation measurement. In-orbit rotation measurement is carried out, and various errors are estimated. Real-time rotation measurement is achieved under a rotation rate that is 15-fold higher than the Earth's rotation rate, with a precision of 29 μrad/s.

The derived expressions for rotation measurement are adapted to the PSI experiment and could be applied to analyze the parameter requirement for more general cases. We take a Hyper-like experiment as an example [12]. The satellite is drag-free controlled and has a polar orbit with a height of 700 km. The frame-dragging effect-induced rotation is calculated to be oscillated with an amplitude of 2 × 10^−14^ rad/s at twice the orbit frequency. A pair of atom gyroscopes with opposite atom velocities are installed in it. The interference time is set at 10 s, the velocity of the atom cloud is set at 0.2 m/s with a precision of 1 μm/s, double diffraction with 4 photon recoil is used to form the interference loop, the signal-to-noise ratio of the interference fringe is set at 10^4^, and the data sampling rate is 1 Hz. Then, the measurement resolution of the rotation is calculated to be 4 × 10^−14^ rad/s/$\sqrt {{\mathrm{Hz}}} $, and a resolution of 7 × 10^−18^ rad/s can be reached for 1 year of data integration. With these parameters, the experiment can measure the frame-dragging effect with a resolution of 0.04%. As illustrated in Equation (1), the angle fluctuation of the Raman laser's mirrors will induce rotation measurement uncertainly. For a frame-dragging effect measurement precision of 0.1%, the requirement of the angle fluctuation of the mirrors at the signal frequency has to be <5 × 10^−11^ rad. This puts a strict constraint on the stability of the mechanical structure of the gyroscope.

The CSSAI can measure rotation in 2D and acceleration in 1D. Increasing the number of Raman laser pairs allows this device to realize inertial measurement with complete vector components. By fusing the measurement data of the AI and the classical accelerometers and gyroscope by using the hybridization schemes [32,33], one can eliminate the deadtime effect and realize a space quantum inertial measurement unit, which can be used for inertial navigation of the spacecraft in orbit or deep space. The main constraints of the measurement precision of the rotation are the relatively short interference time with *T* = 75 ms and low effective atom velocity. For longer interference time, the signal-to-noise ratio of the interference fringe is reduced, and the resolution of the rotation measurement is not improved significantly. The main reason is that the non-ultracold atom cloud is used in our experiment, so the atom number loss is large for a long evolution time with the finite imaging area. These could be improved by preparing the atom cloud with ultracold temperatures using evaporative cooling and adiabatic cooling methods [14–21,34,35] and preparing the atom cloud with a reasonable non-zero velocity using the Bloch or Bragg coherent acceleration method [36,37].

The CSSAI also has the capability of measuring acceleration and differential acceleration. Potential applications cover Earth gravity field measurement [38–40], equivalence principle (EP) tests [41,42], gravitational wave detection [43,44], dark matter detection [45,46] and the testing of general relativity effects [45]. The problem of large phase fluctuation caused by vibration could be solved by using hybridization measurement with a classical accelerometer [32,33]. One can extract the in-orbit gravity acceleration by measuring the in-orbit residual acceleration with AI and combining it with the motion acceleration measured by the GNSS [47]. This could be used to invert or examine the gravity model of the Earth. The payload can measure the acceleration of the rubidium isotope synchronously, and the measured acceleration difference forms a quantum test of the EP in space [27,48,49]. Many noise and offset terms could be commonly rejected for the differential measurement, including the spacecraft's residual acceleration and the Raman laser's angle uncertainty, thus increasing the EP test precision.

## MATERIALS AND METHODS

### Time sequence and parameters during the atom interference process

**Figure 4.**
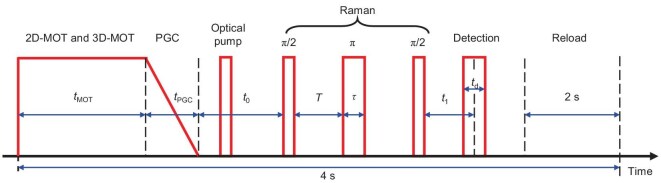
Illustration of the laser intensity variation during the time sequence of the atom interference process of the CSSAI.

The time sequence of the atom interference is shown in Fig. 4. The cycle time is 4.0 s for each experiment. The first *t*_mot_ = 1.4 s is the cooling stage for the 2D-MOT and 3D-MOT. The frequencies of the cooling lasers of the 2D and 3D cooling laser are both −15 MHz detuning to the |5^2^*S*_1/2_, *F* = 2>→|5^2^*P*_3/2_, *F'*=3 > transition and their diameters are both 1.2 cm. The repumping lasers are created by their +1-order sideband, whose powers are ∼50% of the powers of the cooling lasers. The 2D-MOT has a 2D+-MOT configuration [25,50]. The power for each laser beam is 28 mW. The pushing beam is reflected by an in-chamber mirror. This mirror has a hole with a diameter of 2 mm to let the cooled atom pass through, which is shown in Fig. 1a. The magnetic field gradient for the 2D-MOT is 8.5 G/cm. The 3D cooling laser beams are created by a single input laser beam and then reflected by a series of mirrors to reduce the power. The power of the incident laser beam is 26 mW, and the magnetic field gradient for the 3D-MOT is 5.5 G/cm. The combined usage of the 2D- and 3D-MOT creates more than 10^8^ cold atoms in 1.4 s. At the end of the MOT stage, the cooling laser for the 2D-MOT is shut down, and the magnetic fields of the 2D- and 3D-MOT are turned off. The frequency of the cooling laser for the 3D-MOT is turned to −120 MHz detuning to the |5^2^*S*_1/2_, *F* = 2>→|5^2^*P*_3/2_, *F'*=3 > transition, and the power of the cooling laser is ramped to zero in *t*_PGC_ = 2.8 ms to realize the polarization gradient cooling (PGC). The PGC process cools the atom cloud to a temperature of 2 μK, and most of the atoms are populated in the |5^2^*S*_1/2_, *F* = 2 > state. Then the cold atoms are pumped to the |5^2^*S*^1/2^, *F* = 1 > state by an optical pumping laser that resonates with the |5^2^*S*_1/2_, *F* = 2>→|5^2^*P*_3/2_, *F'*=2 > transition. This laser pulse has a power of 46 mW and a width of 20 μs. Then, three Raman laser pulses act on the cold atoms to realize the inference loop, as shown in Fig. 1b. The diameter of the Raman laser is 2.0 cm, the power is 46 mW and the width of the Raman π pulse is *τ* = 17 μs. The two-photon detuning of the Raman laser is set to 74 kHz detuning to its central transition frequency and the interference time is *T*. A constant magnetic field with an intensity of 505 mG is added along the Raman laser. After the interference, the cooling laser beam acts as the detection laser beam to induce the fluorescence of the atom cloud. The frequency of the detection laser pulse is in resonance with |5^2^*S*_1/2_, *F* = 2>→|5^2^*P*_3/2_, *F'*=3 > transition with a width of *t*_d_ = 200 μs. After the fluorescence detection, the main control circuit of the CSSAI will reload the data for the interference time sequence and start the next cycle for the atom interference experiment. This stage lasts for ∼2 seconds.

### Derivation of the exact formulas of the phase and spatial frequency of PSI

As illustrated in Fig. 1c. The position of the atom has the following relationship:


(5) \begin{eqnarray*}
{R_i} &=& {r_i} + {v_i}\!\left( {2T + {t_1}} \right)\\
&=& {\rho _i} + {v_i}t,
\end{eqnarray*}

where *ρ_i_* is the atom's position at the time of MOT release, *t* = *t*_0_ + 2*T* + *t*_1_ represents the total time, and *t*_0_ is the time interval between the release of the MOT and the first Raman pulse. The distribution of the cold atom cloud at the time of MOT releasing is


(6) \begin{eqnarray*}
F\!\left( {{\rho _i},{v_i}} \right) = {N_1}{e^{ - \frac{{{{\left( {{\rho _i} - {\rho _{i0}}} \right)}^2}}}{{2{\sigma _{\rho i}}^2}}}}{e^{ - \frac{{{{({v_i} - {v_{i0}})}^2}}}{{2{\sigma _{vi}}^2}}}},
\end{eqnarray*}

where *ρ*_0_ and *σ_ρi_* are the central and the distribution widths of the position, *v_i_*_0_ and *σ_vi_* are the central and the distribution widths of the velocity, and *N*_1_ is the normalization factor. Then, we substitute Equation (5) into Equations (1) and (6), and eliminate the variables *r_i_* and *ρ_i_*. The population of the atom at the detection time can be calculated by integrating over *v_i_* with the following formula:


(7) \begin{eqnarray*}
&&{P_I}\!\left( {{R_i}} \right)\\
&& = \mathop \int \nolimits_{ - \infty }^\infty P\!\left( {{R_i}\! -\! {v_i}\!\left( {2T\! +\! {t_1}} \right),{v_i}} \right)F\!\left( {{R_i}\! -\! {v_i}t,{v_i}} \right)d{v_i}.\\
\end{eqnarray*}

We find the analytic expression of the integrated phase *ϕ_I_* from the integrated population *P_I_*(*R_i_*), which has the form


(8) \begin{eqnarray*}
{\phi _I} &=& {\phi _o} + {k_{{\mathrm{eff}}}}\mathop \sum \limits_i {\delta _i}\left( {\frac{{{t_0}}}{t}\! +\! \frac{{t\! -\! {t_0}}}{t}\frac{{{\sigma _{\rho i}}^2}}{{{\sigma _{vi}}^2{t^2}\! +\! {\sigma _{\rho i}}^2}}} \right){R_i}\Delta {\theta _j}\\
&& +\, {k_{{\mathrm{eff}}}}\mathop \sum \limits_i {\delta _i}\frac{{t - {t_0}}}{t} \cdot \frac{{{\sigma _{vi}}^2{t^2}{\rho _{i0}} - {\sigma _{\rho i}}^2{v_{i0}}t}}{{{\sigma _{vi}}^2{t^2} + {\sigma _{\rho i}}^2}}{\mathrm{\Delta }}{\theta _j}.\\
\end{eqnarray*}

Besides the optimized phase *ϕ_o_*, the integrated phase is additionally related to the time parameters *t*_0_ and *t*, the parameters of the cold atom cloud *ρ_i_*_0_, *σ_ρi_, v_i_*_0_ and *σ_vi_*, and the difference angle Δ*θ_j_*. The spatial frequency of the integrated phase can be calculated as *f_i_* = ∂*ϕ_I_*/∂*R_i_*. We define it as the integrated spatial frequency, which has the following expression:


(9) \begin{eqnarray*}
{f_i} &=& {f_{io}} + \Delta {f_i}\\
&&{f_{io}} + {k_{{\mathrm{eff}}}}\!\left( {\frac{{{t_0}}}{t} + \frac{{t - {t_0}}}{t}\frac{{{\sigma _{\rho i}}^2}}{{{\sigma _{vi}}^2{t^2} + {\sigma _{\rho i}}^2}}} \right)\Delta {\theta _j}.\\
\end{eqnarray*}

This is the exact formula for the PSI's spatial frequency. Besides the optimized spatial frequency *f_i_*_0_, the integrated spatial frequency is additionally related to the differential angle Δ*θ_j_*, and this formula is used to calculate the rotation *Ω_j_* from the fitted value of *f_i_*. The measurement uncertainty of *Ω_j_* can be calculated by


(10) \begin{eqnarray*}
d{\Omega _j} = \left( {1\bigg/\frac{{\partial {f_i}}}{{\partial {\Omega _j}}}} \right)d{f_i} - \mathop \sum \limits_k \left( {\frac{{\frac{{\partial {f_i}}}{{\partial {p_k}}}}}{{\frac{{\partial {f_i}}}{{\partial {\Omega _j}}}}}} \right)d{p_k},
\end{eqnarray*}

where *df_i_* is the fitting uncertainty of the spatial fringe, and *p_k_* and *dp_k_* represent the parameters and their uncertainties in the expression of *f_i_*.

### Phase modification caused by the finite laser pulse effect

The effect of the pulse width of the Raman laser is not considered in Equation (1). This effect has to be considered for an accurate rotation measurement. The modified phase *ϕ_m_* can be calculated by the sensitive function integrating method [51]. First, the time-dependent frequency and phase responses of the acceleration, rotation and Raman laser's angle are derived, and then their corresponding sensitive functions are calculated. By multiplying these terms and integrating them over time, the expression of *ϕ_m_* is as follows:


(11) \begin{eqnarray*}
{\phi _m} &=& {k_{{\mathrm{eff}}}}{a_z}\!\left( {T + \tau } \right)\left( {T + \frac{{2\tau }}{{\mathrm{\pi }}}} \right)\\
&& +\, {k_{{\mathrm{eff}}}}\mathop \sum \limits_i {\delta _i}2{\Omega _j}{v_i}\left( {T + \tau } \right)\left( {T + \frac{{2\tau }}{{\mathrm{\pi }}}} \right)\\
&& +\, {k_{{\mathrm{eff}}}}\mathop \sum \limits_i {\delta _i}{\theta _{j,1}}\left[ {{r_i} + \frac{{\left( { - 2 + {\mathrm{\pi }}} \right){v_i}\tau }}{{2{\mathrm{\pi }}}}} \right]\\
&& +\, {k_{{\mathrm{eff}}}}\mathop \sum \limits_i {\delta _i}{\theta _{j,3}}\left[ {{r_i}\! +\! {v_i}\frac{{\left( {4{\mathrm{\pi }}T \!+\! 2\tau\! +\! 3{\mathrm{\pi }}\tau } \right)}}{{2{\mathrm{\pi }}}}} \right],\\
\end{eqnarray*}

where τ is the width of the Raman π pulse. By submitting *r_i_* = *R_i_* − *v_i_*(2*T* + 2τ + *t*_1_) in Equation (11) and letting the coefficient of *v_i_* be zero, we find the optimized angle *θ_jmo_*_,1_ for the modified phase, which has the form


(12) \begin{eqnarray*}
{\theta _{jmo,1}}\! =\! \frac{{ {-}\left( {{t_1}\! -\! \frac{\tau }{{\mathrm{\pi }}}\! +\! \frac{\tau }{2}} \right){\theta _{j,3}}\! +\! 2{\Omega _j}\!\left( {T\! +\! \tau } \right)\left( {T\! +\! \frac{{2\tau }}{{\mathrm{\pi }}}} \right)}}{{2T\! +\! {t_1}\! +\! \frac{\tau }{{\mathrm{\pi }}}\! +\! \frac{{3\tau }}{2}}}.
\end{eqnarray*}

Following the similar calculating process of Equations (7)–(9), one can derive the modified formulas of the integrated phase *ϕ_I_* and integrated spatial frequency *f_i_* of *ϕ_m_*. These modified formulas are used to calibrate the Raman laser's angle and calculate the rotation. The formulas are not listed here because of their complex expressions.

## Supplementary Material

nwaf012_Supplemental_File
