# Supplementary material for: Realization of a cold atom gyroscope in space
Source: Natl Sci Rev. 2025 Jan 11;12(4):nwaf012. doi: 10.1093/nsr/nwaf012 (PMC11960097; doi:10.1093/nsr/nwaf012)
Supplement: nwaf012_Supplemental_File [file nwaf012_supplemental_file.docx]

**Supplementary materials for “Realization of a cold atom gyroscope in space”**

Jinting Li^1,2^, Xi Chen^1^*, Danfang Zhang^1,2^, Wenzhang Wang^1,2^, Yang Zhou^1,2^, Meng He^1^, Jie Fang^1^, Lin Zhou^1,3^, Chuan He^1^, Junjie Jiang^1,2^, Huanyao Sun^1^, Qunfeng Chen^1^, Lei Qin^1^, Xiao Li^1^, Yibo Wang^1^, Xiaowei Zhang^1^, Jiaqi Zhong^1,3^, Runbing Li^1,3,5^, Meizhen An^4^, Long Zhang^4^, Shuquan Wang^4^, Zongfeng Li^4^, Jin Wang^1,3,5^† and Mingsheng Zhan^1,3,5^‡

^1^State Key Laboratory of Magnetic Resonance and Atomic and Molecular Physics, Innovation Academy for Precision Measurement Science and Technology, Chinese Academy of Sciences, Wuhan 430071, China

^2^School of Physical Sciences, University of Chinese Academy of Sciences, Beijing 100049, China

^3^Hefei National Laboratory, Hefei 230088, China

^4^Technology and Engineering Center for Space Utilization, Chinese Academy of Sciences, Beijing 100094, China

^5^Wuhan Institute of Quantum Technology, Wuhan 430206, China

Email: [chenxi@apm.ac.cn](mailto:chenxi@apm.ac.cn), wangjin@apm.ac.cn, mszhan@apm.ac.cn

I. Introduction of the payload and its operation

The CSSAI contains the physical, optical, and electronic systems. The physical system is for cooling and interference of atoms. Its core component is a vacuum chamber with a vacuum level of 1×10^-8^ Pa. An IMU is installed inside the physical system to monitor acceleration and rotation. The optical system provides lasers for atom manipulation. Lasers are coupled to three single-mode polarization-maintaining (SMPM) fibers and sent to the physical system, acting as the lasers for 2D-MOT, 3D-MOT, and Raman interference, respectively. The electronic system drives and controls the payload. Detailed introductions of the payload are provided in Ref. 1. The payload is operated in remote mode. Operational commands are sent to the payload from the Payload Operation and Application Center (POAC) of the CSS on the ground. The experimental data are transmitted from the payload to the POAC for real-time data monitoring and post-data processing.

The CSSAI is installed inside the Free-floating Platform for Microgravity Experiment (FPME) of the High Microgravity Level Research Rack (HMLR) in the CSS, as shown in Fig. 1A. The FPME has a two-layer structure. The positions and attitudes of the two layers are controlled by gas thruster and magnetic field coils, respectively. This platform can provide a microgravity environment of 10^-7^ g level. The CSSAI is installed on the mounting board of the inner layer. So, the payload has no effective heat-dissipation channel, and the temperature rises when it operates, as shown in Fig. S1B. To maintain the stability of the payload’s parameters and protect the payload from overheating, the CSSAI is operated intermittently. Each experiment lasts for 50-70 min, and the temperature rises for about 6 ˚C. In the first 10 min, the payload is turned on. The temperatures of the lasers and Rb vapor cells are controlled, and the frequencies of seed lasers are auto-locked. The vacuum of the physical system can be stabilized to 10^-8^ Pa during this stage, as shown in Fig. S1C. In the next 20 min, the preset atom interference experiment time sequence is executed periodically. In the last 20-40 min, the payload is operated in a standby mode to minimize the power consumption. Experiment data are transmitted to the CSS’s host computer. Then, the payload is powered off and naturally cooled to the room temperature of the CSS for 3-4 hours.


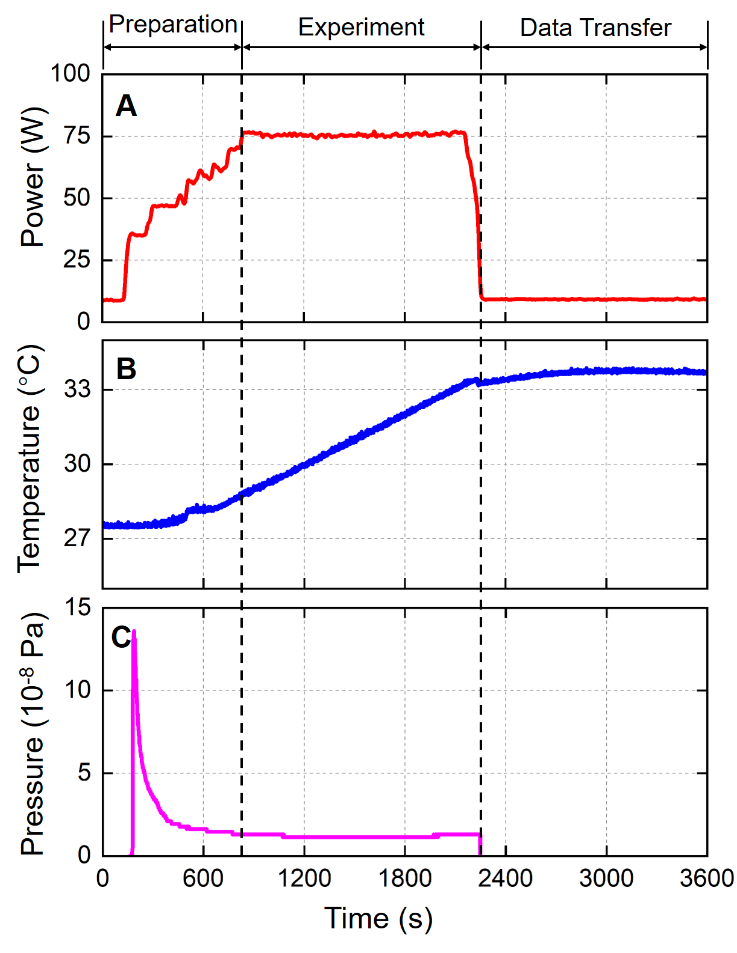


**Figure S1.** Parameters variation when CSSAI operated in orbit. (A) Power of the payload. (B) Temperature of the optical system. (C) Vacuum degree of the physical system.

For the rotation measurements in this article, the FPME is in a fixed-connecting state. The vibration noise is much higher than the free-floating state which is mainly caused by the vibration of the racks installed in the CSS. The comparation of vibration measured by the FPME between these two states are shown in Fig. S2. Under the fixed-connecting state, large vibration acts on the CSSAI, and this is the reason that the measure phase variation of the interference fringe exceeds 2π, as shown in Fig. 3B.


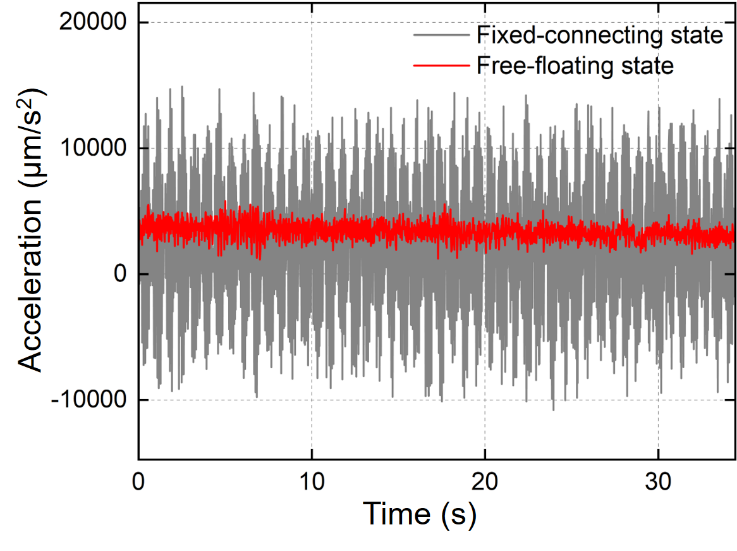


**Figure S2.** Vibration acceleration that the CSSAI felt under the fixed-connecting state and free-floating state.

II. Determine the magnification factor for the imaging system

The spatial interference fringes are generated using the PSI method. After interference, the fluorescence of atoms in the |5^2^S_1/2_, F=2> state is excited by the detection laser. The fluorescence is then collected by the imaging system and imaged by the SCMOS camera. To accurately extract the period of the interference fringes from the image, the imaging system's magnification factor has to be evaluated. The real and the imaged fringe frequencies have the following relationship.

$f_{i}=f_{i,p}/\kappa$, (S1)

where $f_{i}$ denotes the real spatial frequency of the fringe in the i-direction, $f_{i,p}$ denotes the spatial frequency of the imaged fringe, and *κ* is the object-image ratio. We designed an imaging system with 4 lenses with high imaging resolution, as shown in Fig. S3. From the simulation result, the resolution is less than 40 μm, and the object-image ratio is 2.22. The imaging system is manufactured and assembled accurately, which guarantees the accordance from the actual performance to the simulation result.

The position of the cold atom cloud might have some offset from the designed object plane, and the atom cloud also has position distribution at the time of fluorescence detection. For the parameters of PSI in this article, the evaluated distribution of the atom cloud does not exceed ±5 mm. The object-image ratio might differ for atoms in different positions along the optical axis of the imaging system. To evaluate this influence, we carried out a calibration experiment to measure the ratio change of the imaging system. A resolution plate is placed at the position of the object plane and can be moved upward and downward. The images of the resolution plate at different positions are shown in Fig. S4. We find that the variation in the object-image ratio does not exceed ±0.03 when the position change of the board is within ±5 mm.


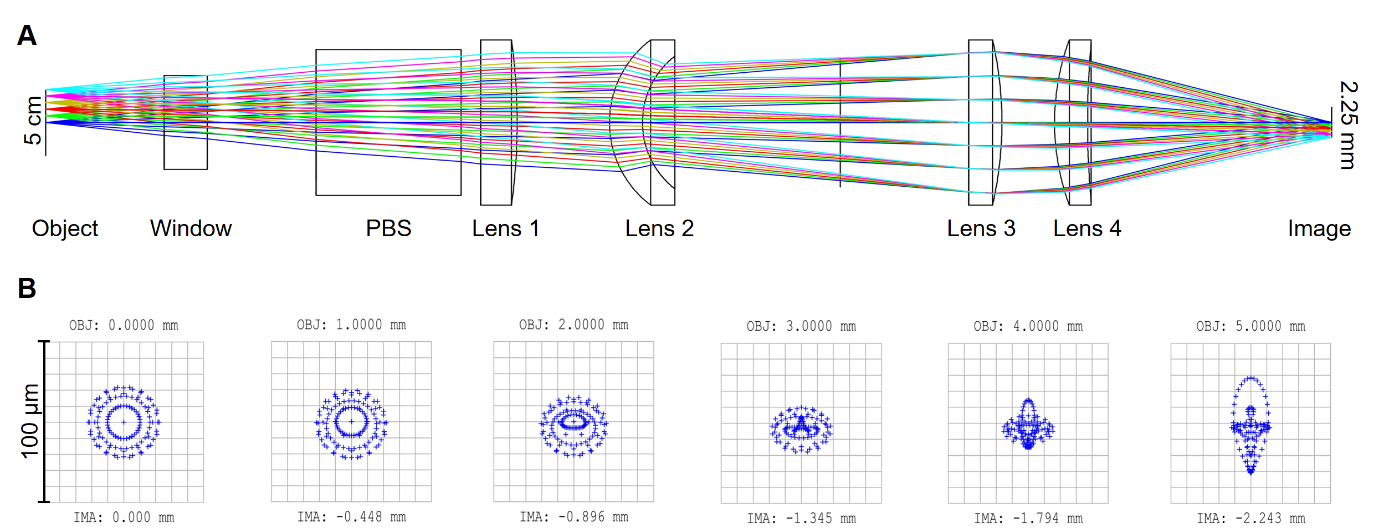


**Figure S3.** The designed imaging system and the simulated spot diagram. (A) Schematic diagram of the imaging system. (B) Spot diagrams of the focal spots with objects in different positions at the object plane.


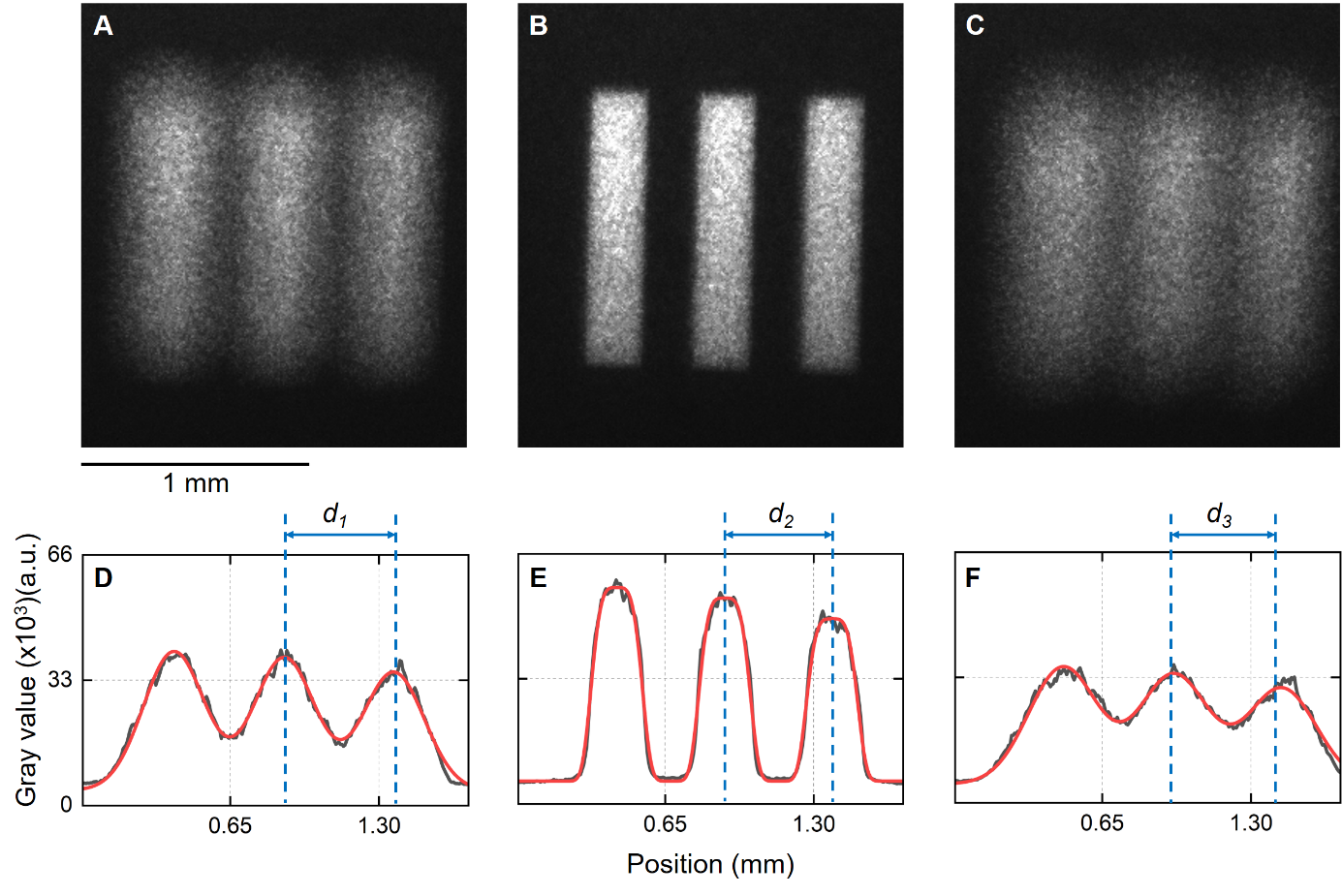


**Figure S4.** Images of the resolution plate at different positions along the optical axis of the imaging system. (A, B, C) Images of the resolution plate with different object distances from its object plane (A: +5 mm, B: 0 mm, C: -5 mm). (E, F, G) averaged and fitted curves of the above images and the measured distances of the fringes. (*d_1_*=0.479 mm, *d_2_*=0.471 mm, *d_3_*=0.477 mm).

III. Method for extracting the spatial fringe from the background

The typical fluorescence image of spatial interference fringe is shown in Fig. S5A. The size of the image is 11 mm×8.2 mm in the x and y direction. After a free evolving time of 233 ms, the full widths at half maximum of the atom cloud are 9.9 mm and 8.0 mm in the x and y direction using 2D Gaussian fitting. The sizes of the atom cloud and the image area are compatible to extract the interference fringe. However, the contrast of the fringe is low, and it is hard to fit the spatial fringe from the 2D image directly. To enhance the signal-to-noise ratio of the spatial interference fringe, the fluorescence image is averaged in an 1D curve. The averaged 1D spatial fringe overlaps with the background of the atomic cloud fluorescence. A typical curve is shown in Fig. S5B. Because of the cold atom cloud's irregular shape, it is difficult to find a suitable formula to fit the curve directly. Therefore, a procedure is designed to normalize the curve to a sine curve before the fitting process. First, the original 1D curves are divided by their Gaussian fitting curves to eliminate the envelopes, and the processed curves are shown in Fig. S5C. The curves fluctuate in offset. The average of these curves is calculated to indicate the offset variation along the *i*-direction. Second, All the processed curves in Fig. S5C are divided by the averaged curve and subtracted by 1, and the processed curves are shown in Fig. S5D. The curves fluctuate in amplitude. The standard derivation of these curves is calculated to indicate the amplitude variation. Finally, the processed curves in Fig. S5D are divided by the standard derivation curve. A typically normalized Sine curve is shown in Fig. S5E.

**
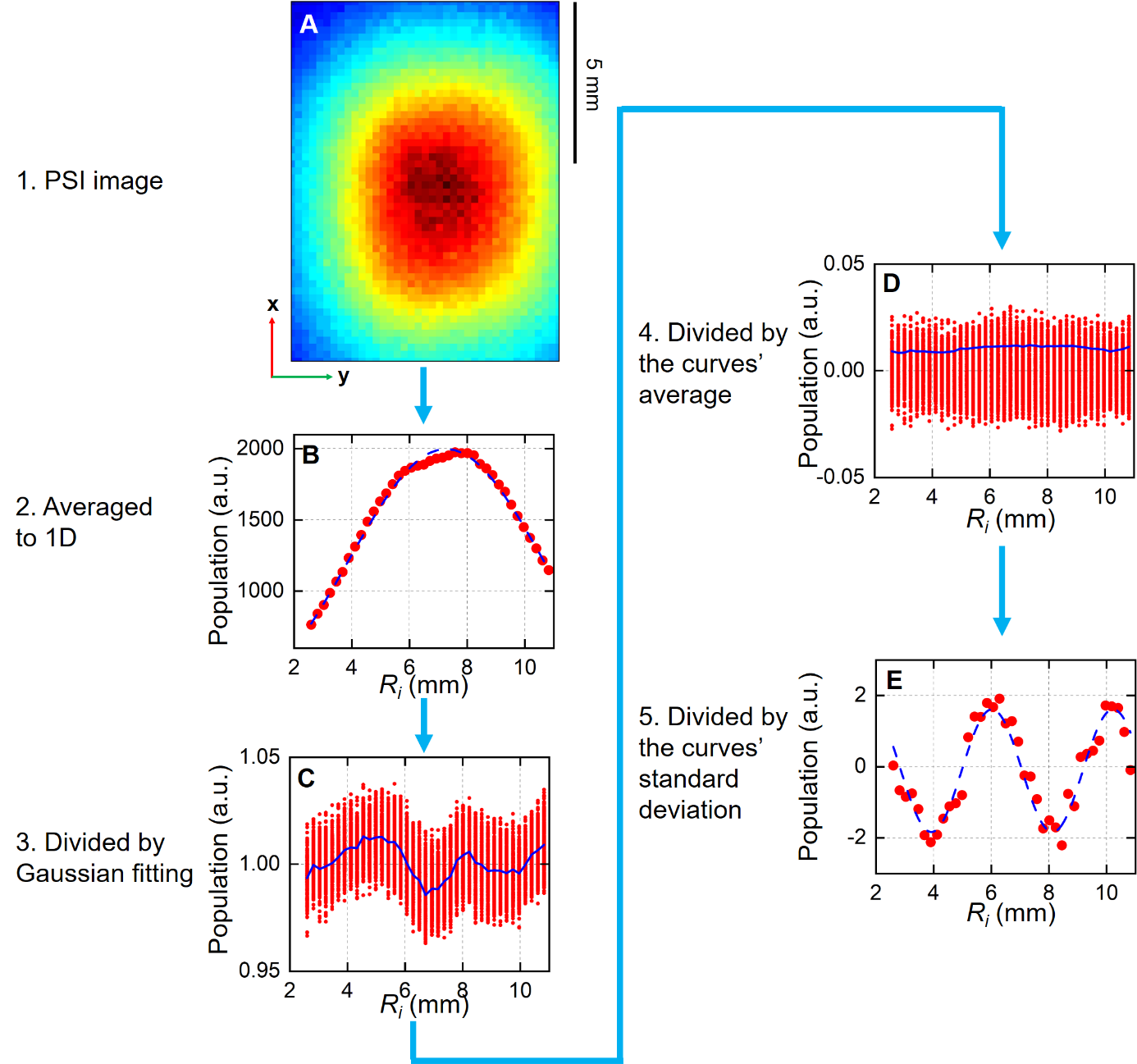
**

**Figure S5.** The normalization method of the PSI fringe. (A-D) The processes from the origin 1D PSI curve with background to the 1D normalized spatial fringe.

A numeral simulation is conducted to check if the above data processing procedures induce offset. The 1D spatial fringe is expressed by the following formula.

$\begin{aligned} P_{Is}=&{N(A}_{n})+H_{A}\left( R_{i} \right)e^{-\frac{\left( R_{i}-R_{i0} \right)^{2}}{2{\sigma_{Ri}}^{2}}} \\ &\times\left[ A_{s}+H_{C}\left( R_{i} \right)C_{s}\cos\left( f_{is}R_{i}+{N(\phi}_{n}) \right) \right] \end{aligned}$, (S2)

where, *H_A_* (*Ri*) and *H_C_*(*Ri*) are polynomial function that represent the offset and amplitude variation, as shown in Fig. S6A. *N*(*u*) is defined as a random number generation function with a zero mean and a standard derivation of *u*. *A_n_* and *ϕ_n_* represent the amplitude of the amplitude noise and phase noise of the spatial fringe. R_i0_ and σ_Ri_ represent the central and distribution width of the Gauss envelope. A_s_, C_s_, and f_is_ represent the set value of offset, amplitude, and spatial frequency. All the simulation parameters are set to close to the practical parameters.

Then, the above data processing procedures are used for the simulated curve. The normalized sine curves are obtained and fitted. The phase and spatial frequency fitting values are compared with the corresponding set values. The differences curves are shown in Fig. S6B and S6C. The phase difference is Δ*ϕ*=16±109 mrad, and the spatial frequency difference is Δ*f_i_*=3±38 mrad/mm. The fitting noise is caused by the introduced amplitude noise. No obvious derivation is found from the fitting values to the set values of the phase and spatial frequency, which means that the proposed data processing procedures introduce no bias.

**
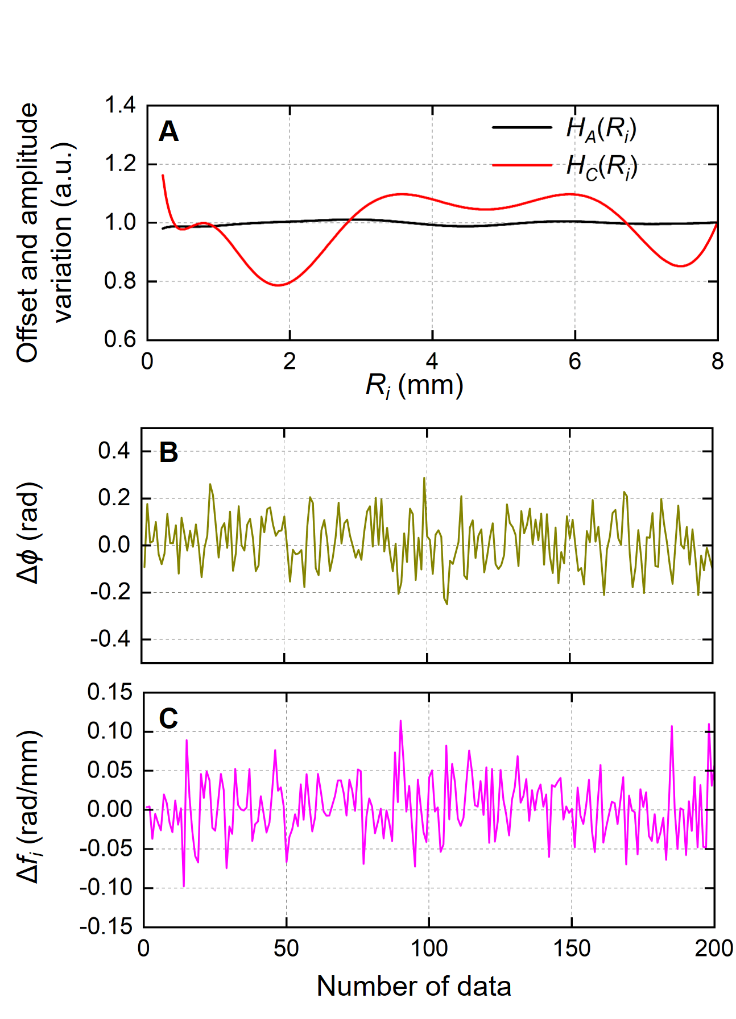
**

**Figure S6.** Numeral simulation of the normalization method. (A) The set profile of *H_A_*(*Ri*) and *H_C_*(*Ri*) for the numeral simulation. Other parameters are set as *A_n_*=0.005, *ϕ_n_*=10 rad, *R_i_*_0_=5 mm, *σ_Ri_*=5 mm, *A_s_*=1, *C_s_*=0.02, *f_is_*=1.52 rad/mm. (B, C) The difference between the fitted and set values of the phase and the spatial frequency of the simulation.

IV. Determine the formula for the interference phase of point sources.

By solving the Lagrange equations, the motion equation of the cloud atom in the CSS is expressed as

$\frac{d^{2}\boldsymbol{r}}{dt^{2}}=\left( \boldsymbol{g}-\boldsymbol{a}_{c} \right)+r_{i}T_{ij}-\frac{d\boldsymbol{\Omega}}{dt}\times\boldsymbol{r}-2\boldsymbol{\Omega}\times\frac{d\boldsymbol{r}}{dt}-\boldsymbol{\Omega}\times\left( \boldsymbol{\Omega}\times\boldsymbol{r} \right)$, (S3)

where ***r*** is the coordinate of the atom in the center of mass coordinates of the CSS. ***g*** and ***a***_c_ are the gravitational acceleration and the centrifugal force of the CSS at the position of its center of mass, *T_ij_* is the gravity gradient tensor, ***Ω*** is the rotation rate of the CSS. By solving the trajectory of the atom of the interference loop using Eq. (S3), the phase of the interference fringe can be obtained by calculating the laser phase and the path integration phase [2], and then expanded into the power series of the interference time *T*. The expression of the phase up to the 3-order of T is

$\begin{aligned} \phi=&k_{\mathrm{eff}}(g_{z}-a_{c}+T_{zz}r_{z}+\sum_{i} {\Omega_{i}}^{2}r_{z})T^{2} \\ &+\sum_{i} {\delta_{i}k}_{\mathrm{eff}}\left( 2\Omega_{j}T_{ii}T^{3}+\frac{d\Omega_{j}}{dt}T^{2}+\theta_{j,1}+\theta_{j,3} \right)r_{i} \\ &+\sum_{i} {\delta_{i}k}_{\mathrm{eff}}(2\Omega_{j}T^{2}+2\theta_{j,3}T+\frac{d\Omega_{j}}{dt}T^{3})v_{i} \end{aligned}$. (S4)

The definitions of *i,j*, and *δi* are the same as Eq. (1). The phase can be divided into 3 parts, as shown in the right side of Eq. (S4). The first term is not related to *r_i_* and *v_i_*. Because the spatial interference image is along the *x* and *y* directions, this term will not influence the spatial frequency of the fringe and, thus, the rotation measurement. We define *a_z_* = *g_z_* – *a*_c_ +*T_zz_r_z_+*∑*_i_Ω_i_*^2^*r_z_* as the residual acceleration as shown in Eq. (1). The second and third terms are related to the *r_i_* and *v_i_*. There are additional terms related to d*Ω_j_*/d*t* and *Ω_j_T_ii_* beside the terms in Eq. (1). For the parameters of our experiment, *T_ii_* is about 1×10^-6^ s^-2^. d*Ω_j_*/d*t* is calculated by the linear fitting result of the rotation measured by the gyroscope of the CSS and is 1.3×10^-8^ rad/s². The three additional terms in Eq. (S4) are calculated to be 6-8 orders smaller than other terms and can be safely omitted. Then Eq. (S4) can be reduced to Eq. (1).

V. Simulation of the spatial fringe

The simulated 1D spatial fringes using Eq. (7) are shown in Fig. S7A- S7C. For Fig. S7A, *θ_j_*_,1_ equals *θ_jo_*_,1_, and the contrast of the spatial fringe is maximum. For Fig. S7B and S7C, *θ_j_*_,1_ deviates from *θ_jo_*_,1_, and the contrast is lower. The obtained curves are fitted with a formula of a sine function plus a Gauss function. The fitted results of the contrast and spatial frequency variation with the change of *θ_j_*_,1_ are shown in Fig. S7D and S7E. For the simulation parameters used in Fig. S7, an offset of *θ_j_*_,1_ of 1 μrad will result in a spatial frequency change of 3.5 mrad/mm and a corresponding rotation measurement offset of 3.6 μrad/s.


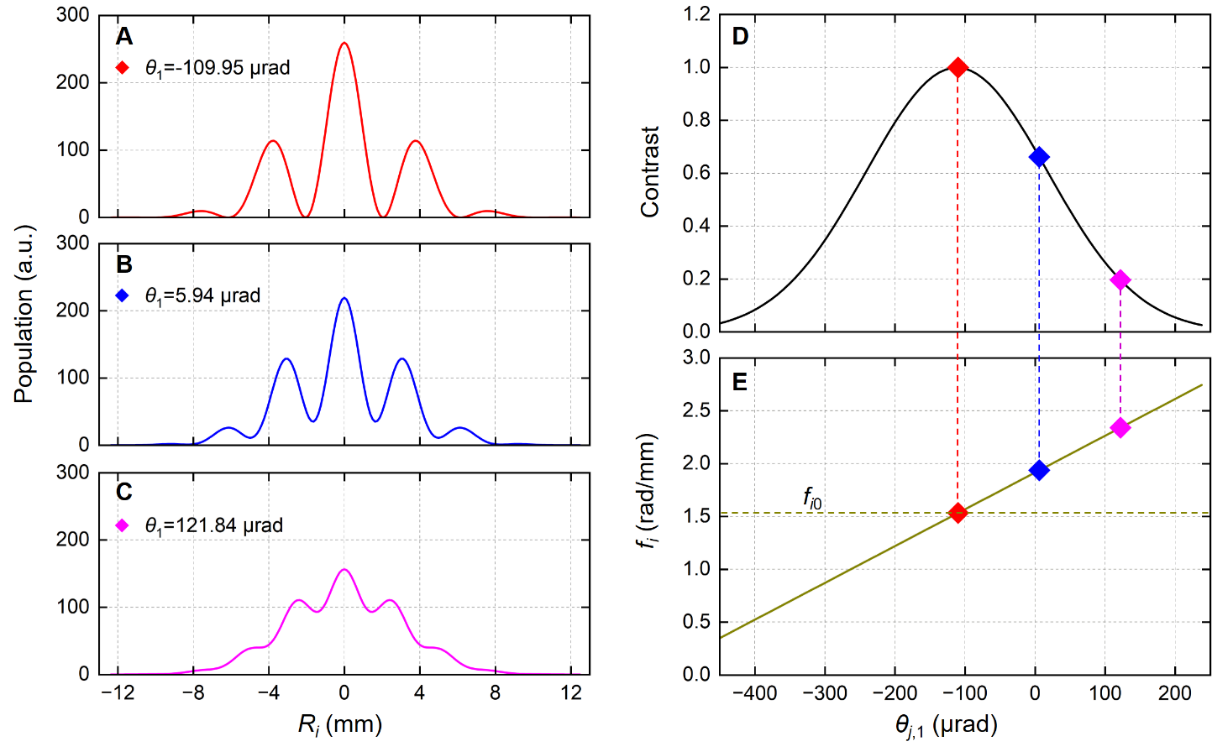


**Figure S7.** Simulation of the 1D spatial interference fringe. (A-C) simulated 1D spatial fringe with different values of *θ_j_*_,1_. Other parameters are *k_eff_*=1.61×10^7^ m^-1^, *a_z_*=0 m/s^2^, *T*=75 ms, *Ω_j_*=-1150 μrad/s, *θ_j_*_,3_=207 μrad, *t*_0_=43 ms, *t*_1_=40 ms, *ρ*_0_=0 mm, *σ_ρi_*=0.6 mm, *v_i_*_0_=0 mm/s, and *σ_vi_*=13 mm/s. (D, E) The fitted contrast and spatial frequency from the simulated fringes. The three pairs of diamonds represent the fitting results of Fig. S7A-S7C.

VI. Error estimation for the rotation measurement

A. The angles of Raman lasers

Equations (4) and (9) show that two angles affect the spatial frequency. One is the angle of the third Raman laser pulse *θ_j_*_,3_, and the other is the difference angle Δ*θ_j_*. For the rotation measurement in Fig. 3, *θ_j_*_,3_ is set to 202.94±0.72 μrad. The uncertainty is caused by the calibration uncertainty of the Raman laser’s angle. Δ*θ_j_* is calculated to be 2.41±0.41 μrad by using the set angle *θ_j_*_,1_, and its optimized angle is calculated by using Eq. (12). These two terms' correction and correction uncertainty are calculated using Eq. (10), and the calculated result is shown in Table 1.

B. The cold atom cloud’s distribution

For the ideal case that Δ*θ_j_*=0, the parameters of the atom cloud do not influence the spatial frequency, as illustrated in Eq. (9). For the experiment for Fig. 3, the set angle *θ_j_*_,1_ has a certain offset from its optimized angle. Hence, the parameters of the cold atom cloud must be evaluated to estimate the correction for the rotation measurement. These include the *σ_ρi_*, and *σ_vi_* from Eq. (9). The fluorescence image at the moment of MOT release and fluorescence detection is recorded as shown in Fig. S8A, and the time of flying (TOF) method is used to measure these two distribution widths. The measured results are shown in Fig. S8B and S8C. The width of position distribution is 0.590 mm and 0.427 mm in the x and y direction, and the width of velocity distribution is 17.04 mm/s and 14.13 mm/s in the x and y direction. The correction and correction uncertainty caused by these two terms are calculated using Eq. (10), and the calculated result is shown in Table 1.

**
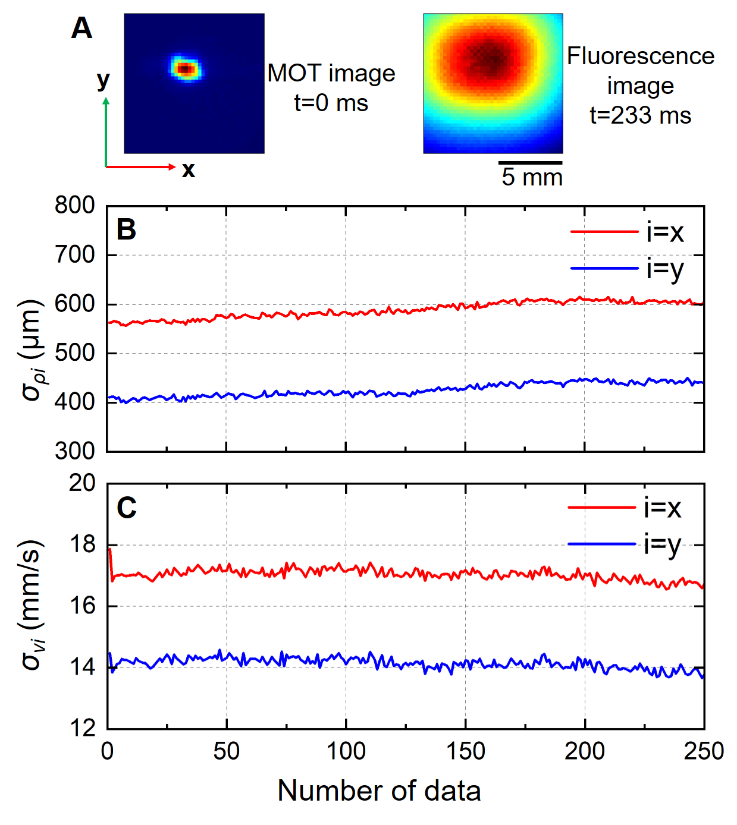
**

**Figure S8.** The measured widths of the position and velocity distribution of the cold atom cloud using the TOF method. (A) The fluorescence image at the moment of MOT release and fluorescence detection. (B, C) Measured widths of the position and velocity distribution of the 3D-MOT.

C. The time uncertainty

Eq. (4) and (9), associated with Method D2, indicate that several terms of the time will influence the spatial frequency of PSA. These include *T*, *t*_0_, *t*_1_, and *τ*, as shown in Fig. 1C. The time sequence is controlled by the FPGA circuit of the CSSAI, which uses a crystal oscillator as the time reference. The oscillator has a time accuracy of 3 ppm. The accuracy of the set values of the time terms can be evaluated by their time reference, and the influence on the rotation measurement is estimated using Eq. (10), and the calculated result is shown in Table 1.

D. The laser frequency uncertainty

The effective wavevector influences the value of the spatial frequency, as illustrated in Eq. (4) and (9). For the CSSAI, the laser frequency is stabilized by using the sideband frequency locking method, and the Raman laser pair is created by phase modulation of a fiber electro-optical modulator (FEOM). The wavevector can be expressed as

$k_{\mathrm{eff}}={2\pi(2\nu}_{a}-{2\nu}_{s}+\nu_{m})/c$, (S4)

where *ν_a_* is the locking frequency of the reference spectral line, *ν_s_* is the modulation frequency for the sideband frequency locking. *ν_m_* is the modulation frequency of the FEOM to create the Raman laser. For the experiment in Fig. 3, The +1 order sideband of the reference laser is locked to the reference frequency *ν_a_* to the transition ^87^Rb |5^2^S_1/2_, *F*=3> -> |5^2^P_3/2_, *F’*=co(3,4)>, The locking uncertainty is about 1 MHz. The *ν_s_* is set to 696.2 MHz. *ν_s_* is determined by a voltage-controlled oscillator (VCO), which has an accuracy of about 2 MHz. The *ν_m_* is set to 6834.8 MHz. *ν_m_* is created by a Phase-locked loop (PLL), which uses an oven-controlled crystal oscillator as a frequency reference. The oscillator has an accuracy of 0.1 ppm. The total uncertainty of the wavevector is 0.09 m^-1^. The influence on the rotation measurement is estimated using Eq. (10), and the calculated result is shown in Table 1.

E. The residual magnetic field

For the rotation measurement, we measured the spatial frequency, which is the phase gradient over space. So, the fixed phase does not influence the spatial frequency measurement. Only the 2^nd^ or higher order of magnetic field gradient can induce the spatial dependence phase shift. The magnitude of the phase shift in the *i* direction can be calculated as

$\phi_{B}=2\pi\hbar k_{\mathrm{eff}}\left( {\alpha_{Rb87}}/{m_{Rb87}} \right)B_{0}\gamma_{i,2}T^{2}R_{i}$, (S5)

where *α_Rb87_* is the second-order Zeeman shift coefficient for the ^87^Rb’s Raman transition, *m_Rb87_* is the mass of the ^87^Rb atom, and *γ_i_*_,2_ is the 2^nd^-order magnetic field gradient in the *i* direction.

The CSSAI uses 3 layers of magnetic shield to shield the external magnetic field, and the residual magnetic field inside the magnetic shield was measured on the ground before the payload launch. The result is shown in Fig. S9. The magnetic field fraction is 0.2 mG. By polynomial fitting this point, the 2^nd^-order magnetic field gradient is found to be -1.3 G/m^2^. In space, the external magnetic field changes periodically, and the residual magnetic field might change with time. However, the ground measurement result can be used to estimate the magnitude of this effect. The induced uncertainty for rotation measurement is estimated using Eq. (10), and the calculated result is shown in Table 1.


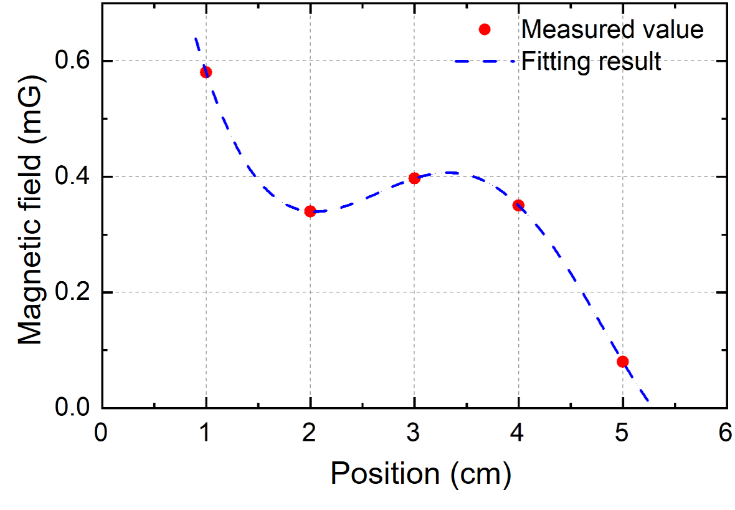


**Figure S9.** The measured residual magnetic field inside the magnetic shield on the ground before the payload launch. Dots represent the measurement result, and the solid line represents the polynomial fitting result.

REFERENCES

1. He M, Chen X, Fang J, et al. The space cold atom interferometer for testing the equivalence principle in the China Space Station. *NPJ Microgravity*. 2023/07/28 2023;9(1):58. doi:10.1038/s41526-023-00306-y
2. Bongs K, Launay R, Kasevich MA. High-order inertial phase shifts for time-domain atom interferometers. *Appl Phys B*. 2006/09/01 2006;84(4):599-602. doi:10.1007/s00340-006-2397-5
